# Ranking of osteogenic potential of physical exercises in postmenopausal women based on femoral neck strains

**DOI:** 10.1371/journal.pone.0195463

**Published:** 2018-04-04

**Authors:** Pim Pellikaan, Georgios Giarmatzis, Jos Vander Sloten, Sabine Verschueren, Ilse Jonkers

**Affiliations:** 1 Biomechanics Section, Department of Mechanical Engineering, KU Leuven, Leuven, Belgium; 2 Research Group for Musculoskeletal Rehabilitation, Department of Rehabilitation Sciences, KU Leuven, Leuven, Belgium; 3 Human Movement Biomechanics Research Group, Department of Movement Sciences, KU Leuven, Leuven, Belgium; University of Zaragoza, SPAIN

## Abstract

The current study aimed to assess the potential of different exercises triggering an osteogenic response at the femoral neck in a group of postmenopausal women. The osteogenic potential was determined by ranking the peak hip contact forces (HCFs) and consequent peak tensile and compressive strains at the **superior** and **inferior** part of the femoral neck during activities such as (fast) walking, running and resistance training exercises. Results indicate that fast walking (5–6 km/h) running and hopping induced significantly higher strains at the femoral neck than walking at 4 km/h which is considered a baseline exercise for bone preservation. Exercises with a high fracture risk such as hopping, need to be considered carefully especially in a frail elderly population and may therefore not be suitable as a training exercise. Since **superior** femoral neck frailness is related to elevated hip fracture risk, exercises such as fast walking (above 5 km/h) and running can be highly recommended to stimulate this particular area. Our results suggest that a training program including fast walking (above 5 km/h) and running exercises may increase or preserve the bone mineral density (BMD) at the femoral neck.

## Introduction

Osteoporosis constitutes a major public health threat, affecting 27.6 million men and women in EU27 in 2010 [1], manifested by bone fractures with an estimated treatment cost up to 37 billion euros. Hip fractures are the most predominant among all osteoporotic-related fractures with the highest morbidity rates [2] in the elderly population. Femoral neck fractures make up approximately 40% - 50% of all hip fractures and occur about three times more often in woman, underlining the sensitivity of this specific region. Exercise interventions such as (brisk) walking are known to increase bone density and strength at the femoral neck, however the osteogenic potential of this specific region during exercise has yet to be determined.

It is well established and first described by Julius Wolff in 1892 [3] that bone adapts its microstructure to its external mechanical environment. Based on this hypothesis, Frost [4] developed a mathematical description of bone adaptation, also known as the mechanostat theory. In this theory, it is stated that bone deforms as a result of mechanical loading expressed in compressive or tensile strains triggering an osteogenic bone response that is regulated by electrochemical signals within the osteocytes network and the extracellular fluid when a certain threshold is reached [5]. Although this threshold value is proposed at around 1500 με [4], this applies only to long mammalian bones and varies between specific bone sites, and is affected by both site-specific loading history and characteristics such as rate, volume and frequency [6,7]. Both tensile or compression strains were considered as equally osteogenic, as they both activate osteogenic stem cells in the bone matrix [8]. Various theoretical and numerical approaches have been developed in the recent decades to model this bone remodeling process driven by mechanical stimuli such as stress, strain and strain energy density [9–11]. Micro damage in the bone matrix, disuse and overloading of the bone are also thought to play an important role in its adaptive response [4].

Thus, further research should aim at identifying the optimal mechanical loading of the bone during general or specific exercises, given the different contact load and muscle forces acting on it. In this respect, specific exercises were found to affect the bone mineral density (BMD) distribution by loading specific bone regions. Due to their high impact profile, weight-bearing exercises, such as brisk walking (5–6 km/h), running and jumping, have been found to increase femoral neck BMD in postmenopausal women [12,13]. Instead, habitual walking (~4 km/h [14]) is not associated with BMD changes in the femoral neck of the elderly [15] and can therefore be considered as a baseline exercise for bone preservation [11,15,16]. Moreover, resistance exercises seem to moderately improve hip BMD when they are hip-targeted and at high intensity [17,18], such as 75–80% of the weight that can be lifted at once (1 repetition maximum). The main driving force behind the changes in BMD are the strain differences at the femoral neck during loading triggering local bone adaptation processes. However, these strains cannot be measured directly due to the invasiveness of the available techniques.

Finite element (FE) analysis combined with loading conditions calculated using musculoskeletal models is a non-invasive technique to calculate the strain distribution at specific bone regions under various loading conditions during several exercises. At present, no study assessed the maximum strains in the femoral neck during specific physical exercises to evaluate their bone remodeling potential. Meanwhile, a strain energy criterion introduced by Huiskes et al [11] has been used by Martelli et al [16] to verify if normal walking, weight-bearing and resistance training exercises can trigger an osteogenic effect in the femoral neck. However, all of the weight-bearing exercises included in this study were only performed by two young females instead of a group of elderly people and literature data was used to simulate the resistance training exercises based on joint torque data at specific angles.

The aim of this study was to evaluate the bone remodeling potential at the femoral neck during various exercises in an elderly study population. Peak tensile and compressive strains were compared and ranked to the strains observed during habitual walking at 4 km/h which is considered as a baseline exercise for bone preservation [11,15,16].

## Methods

Data from 14 post-menopausal elderly women (63.9 ± 7 years old) recruited among the local community was collected during several physical activities in three separate sessions as approved by the local Ethics Committee of UZ/KU Leuven (OG032/ ML10444 (s56405)). Post-menopausal woman are the main target group for most training programs that address osteoporosis. Participants with prior conditions that might limit their functional condition such as pain, lower limb fractures or fractures were excluded from the study. A written informed consent was given by all participants. Firstly participants were asked to walk and run on a split-belt treadmill (Forcelink, Culemborg, The Netherlands) from 3 km/h until their highest attainable speed with a self-selected transition speed from walking to running. Whereas all 14 subjects walked at 3–4 km/h, only 7 subjects reached a walking speed of 6 km/h. Running was more demanding; 4 subjects ran at 5 km/h, 8 subjects at 7 km/h and only 5 reached the highest running speed of 9 km/h. Ground reaction forces were recorded through embedded force plates in the treadmill at 1000 Hz and filtered at 6 Hz. More details can be found in Giarmatzis et al. 2017[19].

Secondly, participants were asked to perform a hopping exercise consisting of three consecutive unilateral jumps on the force plate (AMTI OR6 Series, Watertown, MA, USA) separated in a landing and propulsion phase. Ground reaction forces were recorded at 1000 Hz and filtered at 100 Hz. Standard sport footwear was provided in both experiments.

Thirdly, 12 out of 14 subjects performed a dynamic hip abduction (HABD)/adduction (HADD) and flexion (HF)/extension (HE) exercise in standing position while keeping their right leg straight, against an external weight equal to 40, 60 and 80% of the 1 repetition maximum (RM) of the maximal lifted weight based on the guidelines from Brown and Weir [20].

**Fig 1:**
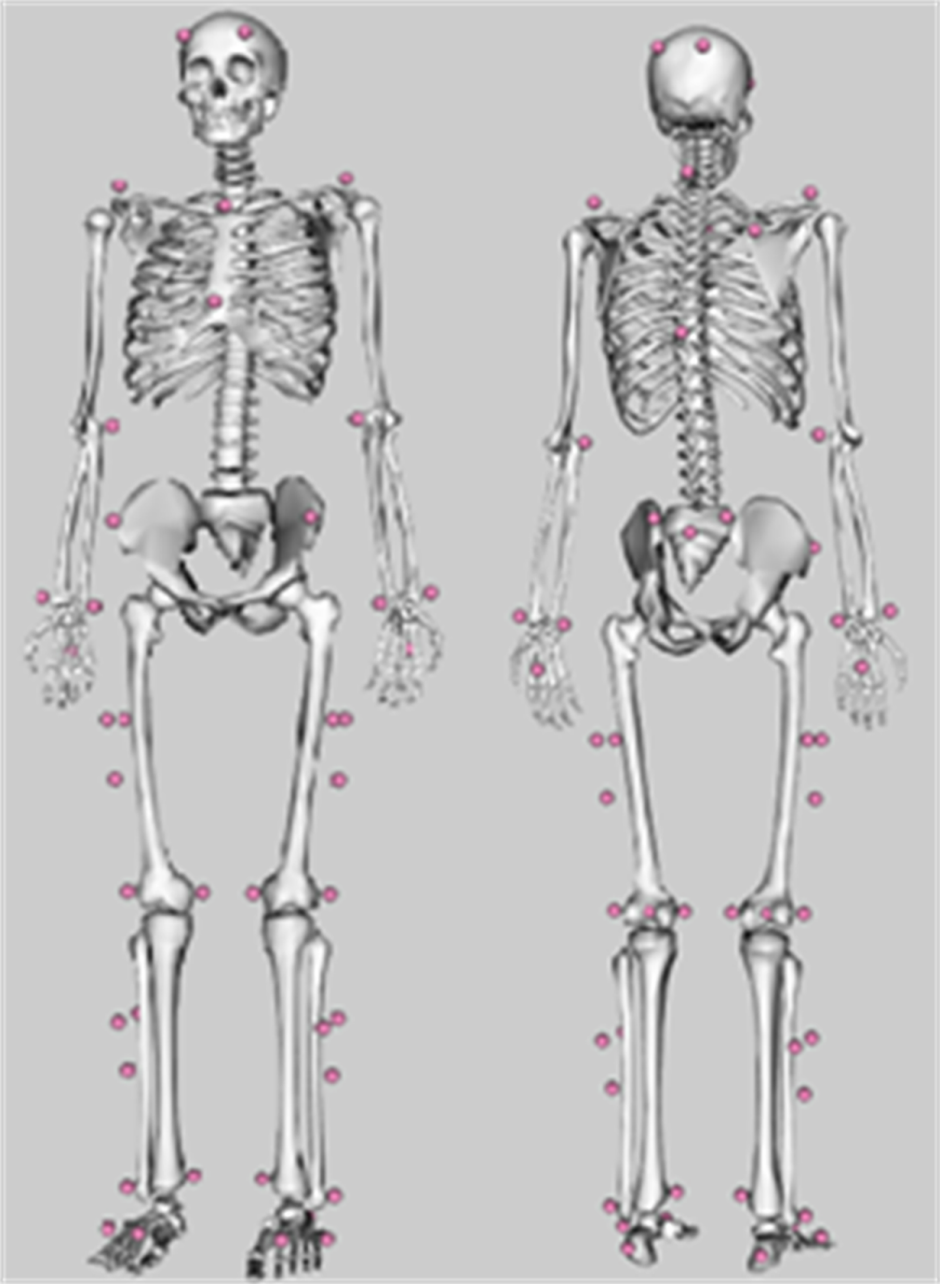
Marker set. Reflective markers were placed on bone anatomical landmarks of the body. Cluster markers were placed on the upper and lower leg for better motion tracking for these specific body segments. During walking, running and hopping the medial markers of the knee and ankle were removed to avoid any adaptation of the natural movement in case of marker contact.

During all exercises, the kinematics were captured using a ten-camera VICON system (10–15 MX camera system, VICON, Oxford Metrics, Oxford, UK) sampled at 100 Hz with an identical marker set [21] (Fig 1).

### Musculoskeletal modeling

A full body generic musculoskeletal model developed by Hamner et al [22] consisting of 12 segments, 29 degrees of freedom (DOF) and 92 Hill-type musculotendon actuators was used. The knee was modeled as a sliding hinge joint, and the ankle as a revolute joint, both presenting one DOF, whereas the hip was modelled as a ball and socket joint with three DOF. Each model was scaled to match the subject’s anthropometric characteristics based on marker data of anatomical landmarks at the hip, knee and ankle during a static trial. Joint angles were calculated using a Kalman smoothing algorithm[23] and muscle forces by static optimization, minimizing the squared sum of all muscle activations. Using a joint reaction analysis, hip and knee contact forces (HCFs and KCFs) were calculated and expressed in the local femur’s coordinate system [24]. Muscle attachment locations and force directions were determined using a dedicated Opensim plugin [25].

### Finite element analysis (FEA)

A finite element model of the femur was constructed in Abaqus (Abaqus 6.14–1, Dassault Systèmes Corp., Providence, RI, USA) identical to the geometry used in the musculoskeletal model. The femur’s geometry was re-meshed to create a volume mesh of 353111 C3D4 tetrahedral elements with a global edge length of 1.5 mm using the Mimics Innovation Suite (Materialise NV, Leuven, Belgium) and Patran (MSC software, Newport Beach, Ca, USA). The edge length was set based on a mesh sensitivity analysis of the maximal stress, strain and strain energy density in the femoral neck region. Hounsfield Units (HU) were determined based on a Femur CT of a healthy 60 year old male with a slice thickness of 1.5 mm and a resolution of 512 by 512 pixels (px) with a size of 0.98mm segmented using the Mimics Innovation Suite (Materialise NV, Leuven, Belgium). The HU’s of the femur CT were used as template and divided in 20 material zones. A linear relation between the HU and bone density as proposed by Bitsakos et al. [26] and Vahdati et al.[27] was assumed and the material properties were assigned to the geometry of the musculoskeletal model using warping techniques that deformed the volume mesh of the musculoskeletal femur geometry to match the geometry of the CT template before assigning the material properties. A material relation defined by Morgan et al. [28] was used to relate the bone mineral density of each material zone to their Young’s Modulus (1400–17500 MPa). The Poisson ratio was set to 0.32 for bone densities above 1.2 g/cc and 0.2 for bone densities below 1.2 g/cc [29].

The location of 26 muscles attachment sites from the generic musculoskeletal model were projected to their closest node point (2.8±1.6mm) on the surface mesh of the FE femur. The pre-defined OpenSim scale factor was used to uniformly scale the volume mesh to each subject’s anthropometry with the femoral head as origin. The muscle parts (#) included in the model were: Gluteus Minimus (3), Medius (3) and Maximus (3), Biceps Femoris Short Head (1), Adductor Longus (1), Brevis (1) and Magnus (3), Pectineus (1), Iliacus (1), Psoas (1), Quadriceps Femoris (1), Gemellus (1), Periformis (1), Vastus Lateralis (1), Medialis (1) and Intermedius (1) and the Gastrocnemius Medialis (1) and Lateralis (1).

Physiological boundary conditions were applied at the center of the femoral head and knee axis and coupled to the surface nodes at the femoral head and lateral and medial condyles respectively similar to Speirs et al. [30]: Boundary conditions were constrained in such a way that the center of the femoral head could only deflect in the direction of the knee axis center without constraining the rotational degrees of freedom. The knee axis was constrained in all directions.

In case of walking, loading conditions at 5 consequence time instances including the first and second peak HCF were determined during a gait cycle and applied to the FE model, including the calculated muscle and joint contact forces. The peak HCF during initial and terminal double support phase, are referred to as the first and second peak of walking. In case of running, loading conditions at 3 consequence time instances including the peak HCF were determined to adequately represent the complete gait cycle. The representative gait or running cycle was selected using the least root mean square error between the HCF curve of each cycle and the average HCF curve of all cycles for each subject and speed. During hopping and resistance training exercises, the instance of subject-specific peak HCF was selected as the only loading condition. For each loading condition the calculated muscle forces were applied to the projected muscle attachment point and distributed over the closest neighboring node points. The hip contact forces were applied to the center of the femoral head and distributed over the femoral head as was done for the boundary conditions.

The superior and inferior part of the femoral neck were analyzed separately as both parts have different bone density distributions and fracture susceptibility, with the superior part being more brittle [31,32]. Therefore, peak tensile (maximal) and compressive (minimal) logarithmic principal strains were calculated at the superior and inferior part of the femoral neck to identify exercises that particularly load these areas of the femoral neck. To do so, the femoral neck was divided in a superior and inferior part along the axis of the femoral neck by determining the anterior/posterior axis using the cross product between femoral neck and the y-axis determined by the ISB local coordinate system of femur [33] and the superior/ inferior axis defined by the cross product of the anterior/posterior and femoral neck axis. In absence of in-vivo strain measurements of the femoral neck, the mean femoral head displacements are reported for comparison with Taylor et al. [34], therefore allowing indirect validation of the model response.

### Statistics

The peak hip contact force and strain in the inferior and superior part of the femoral neck was calculated and averaged for each subject and exercise (S1 Table). The Anscombe variance stabilizing transformation was applied to transform the data into an approximately standard Gaussian distribution. Linear mixed models were constructed with the exercise as a fixed-effect and the variability as a random intercept to account for differences between subjects. These models were used to compare the peak hip contact force and strain for the inferior and superior part of the femoral neck for all exercises. For each linear mixed model a marginal ANOVA test was performed to test the global effect of the exercises on the peak hip contact force and principle strains.

Walking at 7 km/h was excluded from the analysis given the subject sample for that speed was limited to 2.

## Results

**Fig 2:**
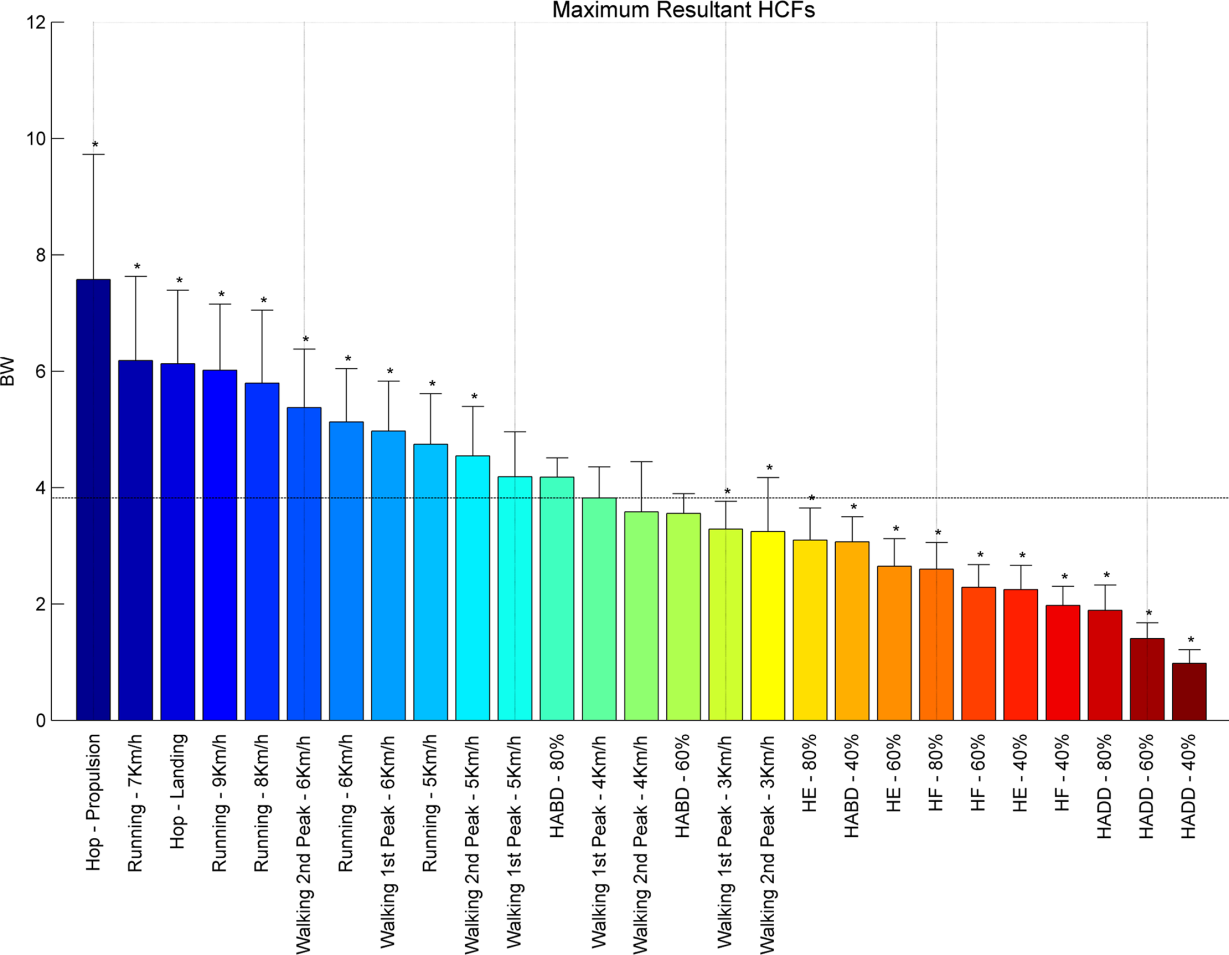
Ranking hip contact forces. Average peak HCFs expressed in body weight of each subject [BW] ranked from left to right for the highest (blue) to the lowest HCF’s (red). Asterisks denote the exercises with significantly different peak HCFs compared to walking at 4 km/h (1^st^ peak) indicated by the horizontal line.

**Fig 3:**
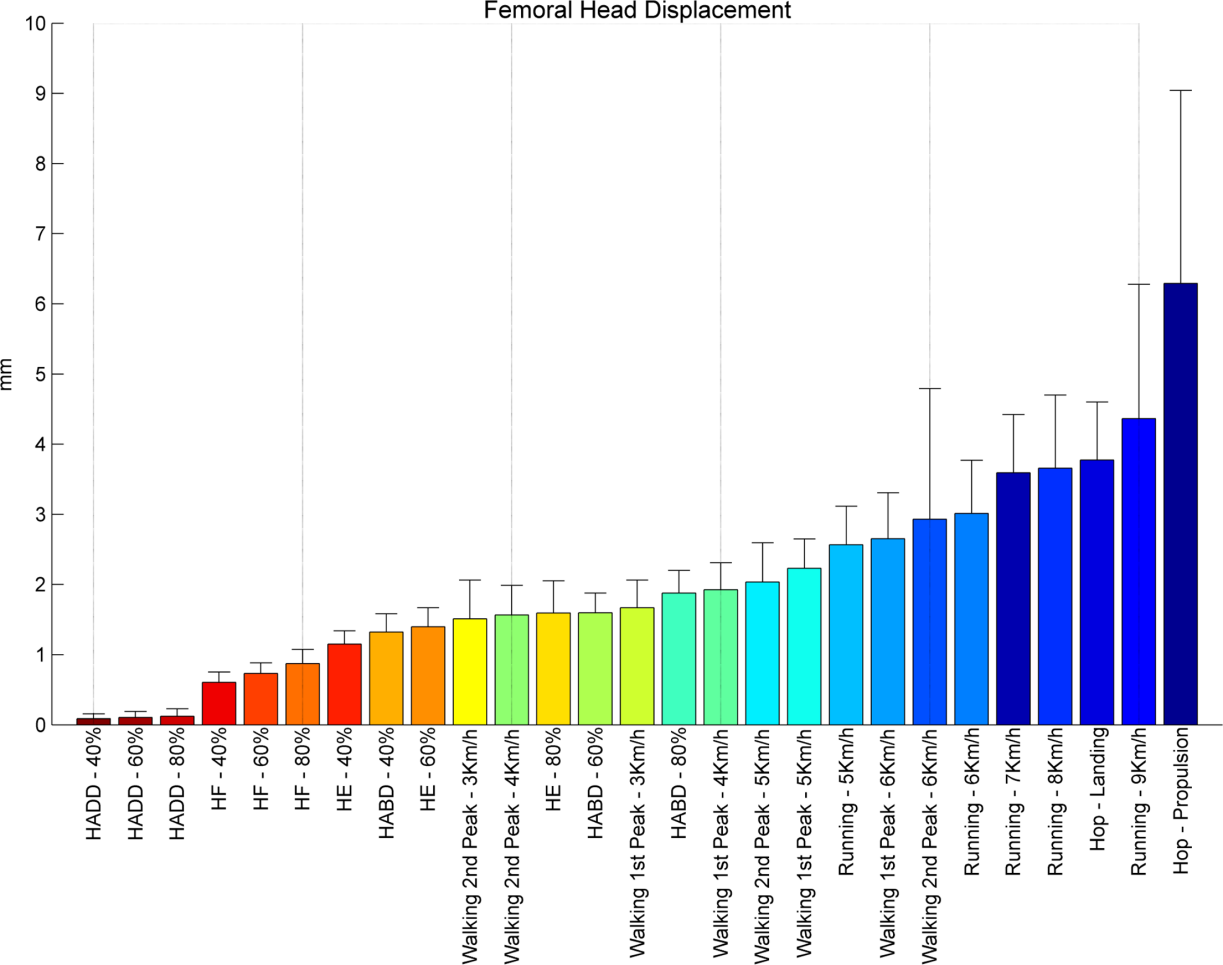
Ranking femoral head displacements. Femoral head displacements in millimeters [mm] during peak HCFs ranked from right to left for the highest (blue) to the lowest displacement (red).

**Fig 4:**
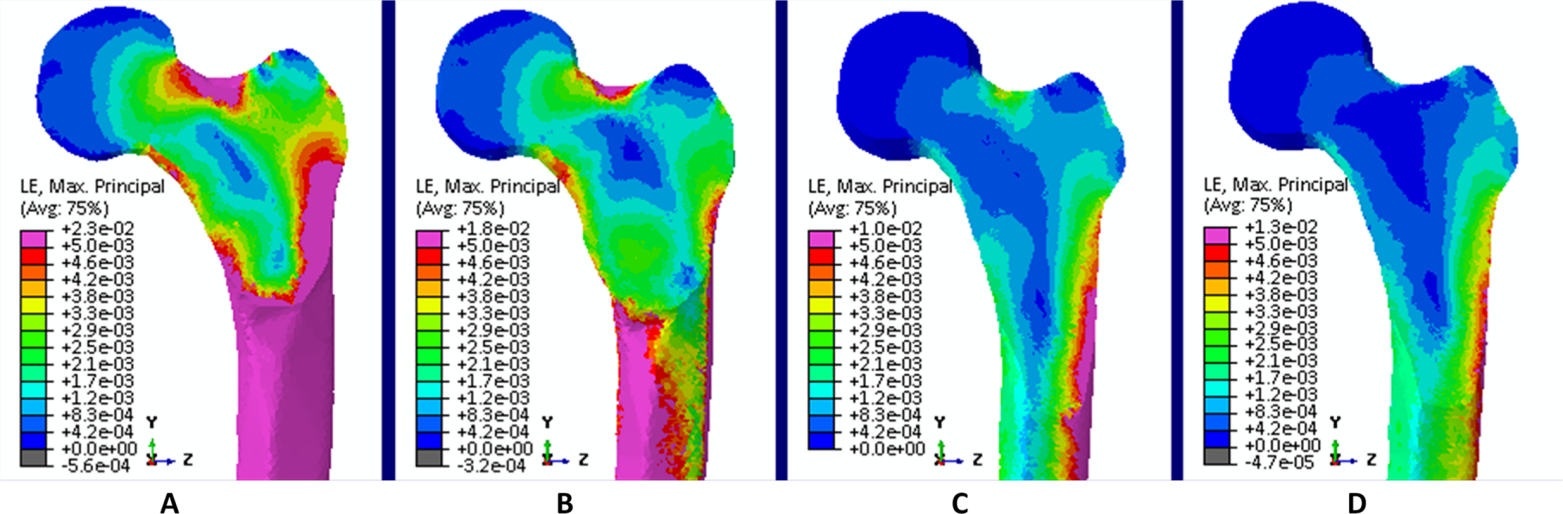
Tensile strains. Tensile strains at the proximal femur for (A) hopping (propulsion), (B) walking 6 km/h (second peak), (C) walking 4 km/h (first peak) and (D) Hip Abduction at 80% RM.

**Fig 5:**
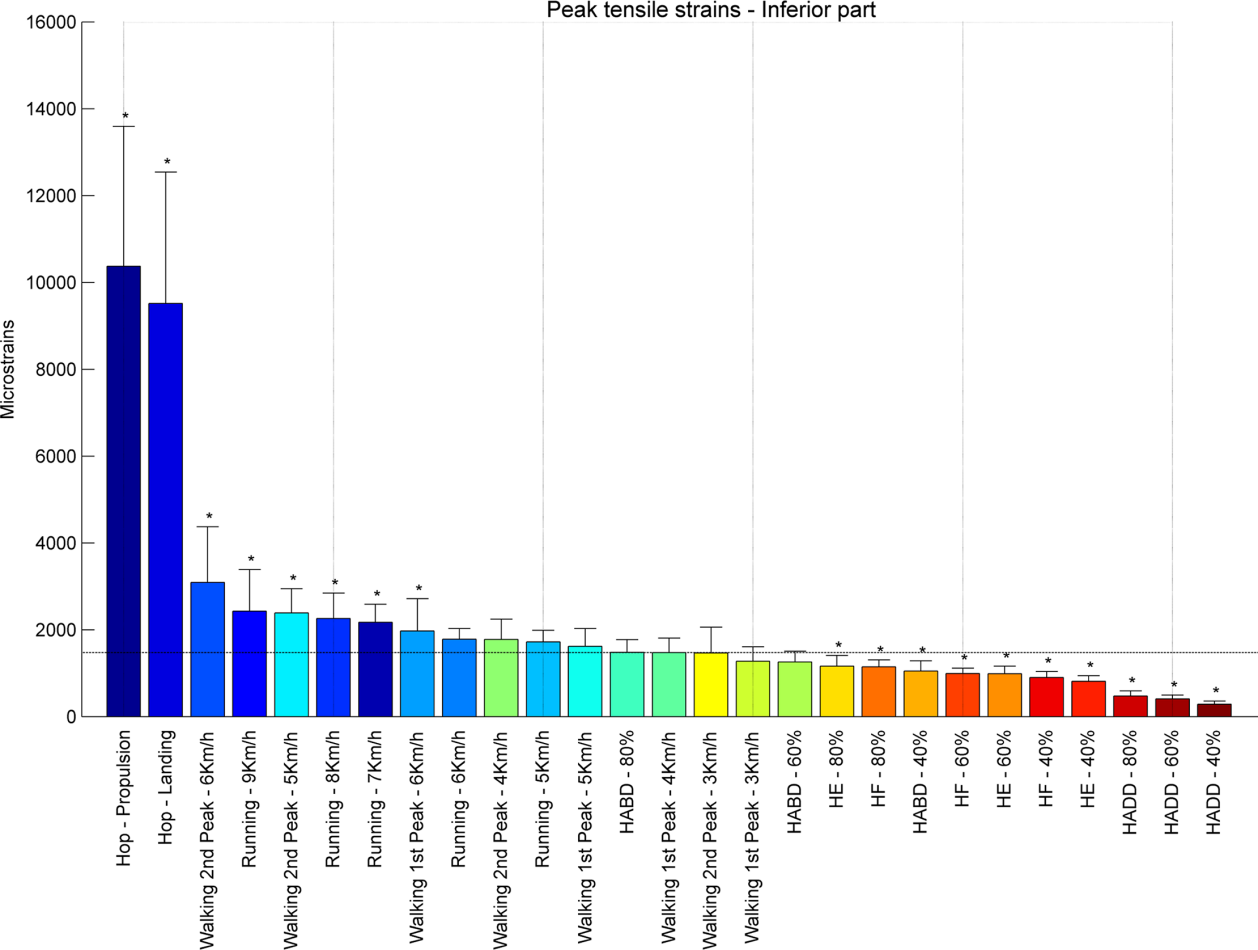
Ranking tensile strains inferior part. Average peak tensile strains in μstrains (εμ) in the inferior part of the femoral neck ranked from left to right for the highest (blue) to the lowest strain (red). Asterisks denote the exercises with significantly different peak tensile strains compared to walking at 4 km/h (1^st^ peak) indicated by the horizontal line.

**Fig 6:**
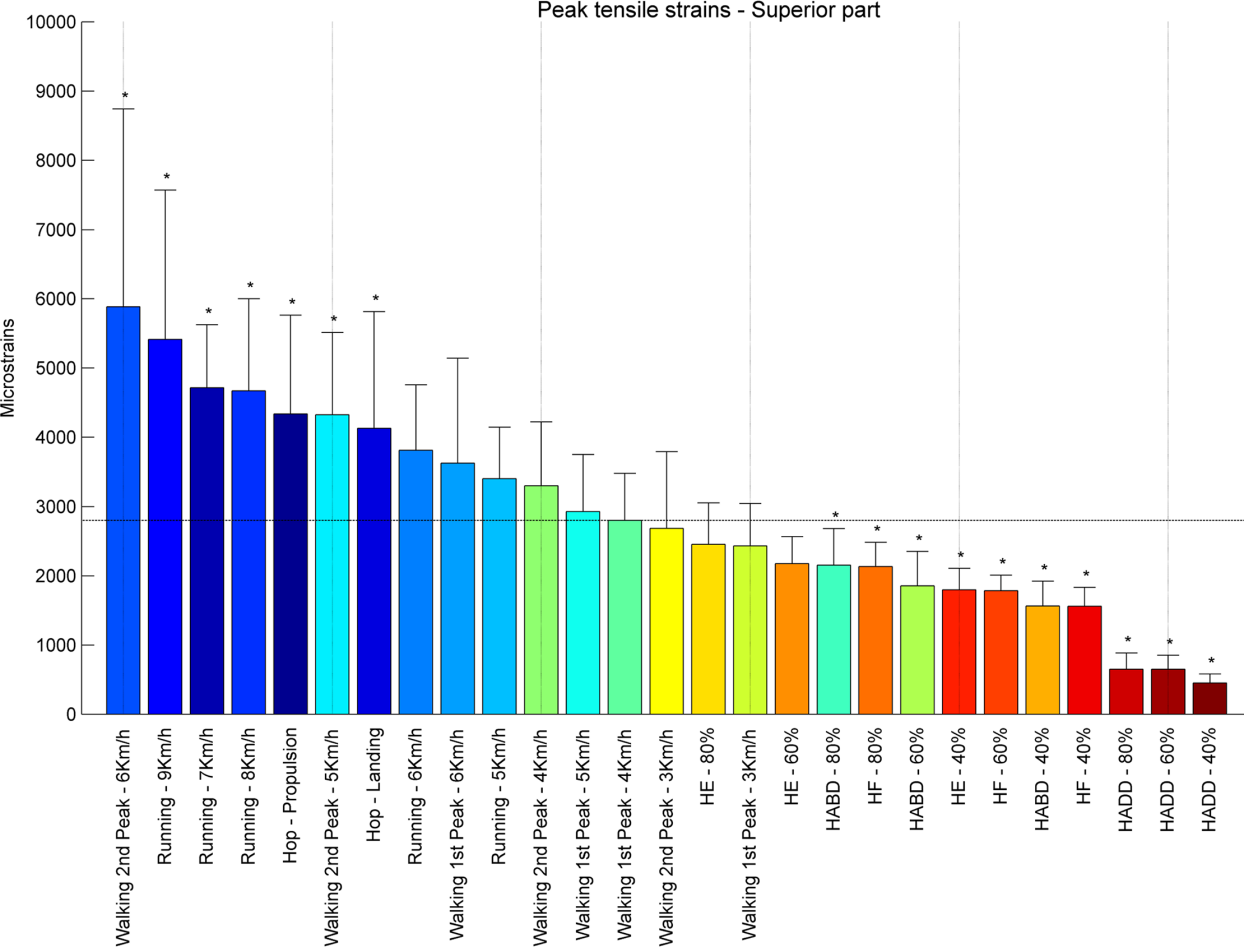
Ranking tensile strains superior part. Average peak tensile strains in μstrains (εμ) in the superior part of the femoral neck ranked from left to right for the highest (blue) to the lowest strain (red). Asterisks denote the exercises with significantly different peak tensile strains compared to walking at 4 km/h (1^st^ peak) indicated by the horizontal line.

**Fig 7:**
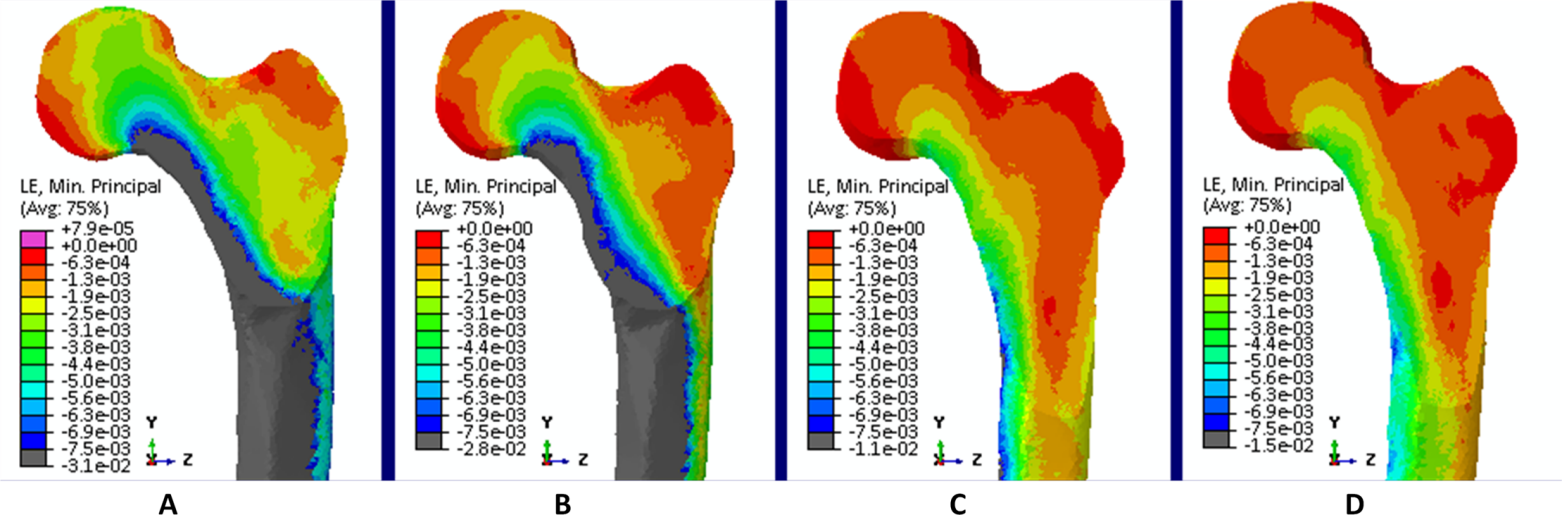
Compressive strains. Compression strains at the proximal femur for (A) hopping (propulsion), (B) walking 6 km/h (second peak), (C) walking 4 km/h (first peak) and (D) Hip Abduction at 80% RM.

Exercises were ranked with respect to the averaged peak HCFs (Fig 2) and the resulting femoral head displacement (Fig 3), peak tensile and compressive principal strains, calculated for the **inferior** and the **superior** part of the femoral neck (Figs 4–7 respectively). Exercises with a significant difference (p < .05) compared to habitual walking at 4 km/h (first peak) were marked with an asterisk. The estimates, lower/upper limits and p-values as well as the results from the ANOVA test for each linear mixed model were reported [S1 Table].

### Hip contact forces

The propulsion phase of hopping imposed the highest peak HCFs (7.57 Body weight (BW)) amongst all exercises, followed by running 7 km/h (6.18 BW), hopping landing phase (6.13 BW), running (8–9 km/h, 5.80 and 6.02 BW respectively) and fast walking (5–6 km/h, 4.55 and 5.38 BW, respectively). Compared to normal walking at 4–5 km/h (3.82 BW and 4.54BW respectively) resistance training exercises loaded the hip less, except for hip abduction at 60% (3.55 BW) and 80% (4.18 BW) of RM, where the HCFs were similar. Overall peak HCFs ranged from 1 BW (hip adduction at 40% 1RM) to 7.57 BW (hop–propulsion) (Fig 2).

### Femoral head displacement

Femoral head displacement during peak load ranged from 1.51 to 2.92 mm during walking and from 2.57 to 4.36 for running (Fig 3). The highest deflection was found during hopping with a displacement equal to 3.78 and 6.29 mm during landing and propulsion respectively. The average displacement for the resistance exercises were 0.10, 0.74, 1.38 and 1.60 mm for respectively hip adduction, flexion, extension and abduction.

### Peak tensile strains

At the **inferior** part of the femoral neck, hopping induced the highest tensile strains, during propulsion (10373 με) and landing (9517 με) (Fig 4). Also running and fast walking imposed higher tensile strains ranging from 1616 με for the first peak of walking at 5 km/h to 3089 με for the second peak of walking at 6 km/h compared to the first peak of walking at 4 km/h (1474 με). All resistance training exercises except for hip abduction at 80% RM (1480 με) induce lower strain values. Overall, peak tensile strains at the inferior part of the femoral neck ranged from 284 με (hip adduction at 40% RM) to 10373 με (hopping–propulsion) (Fig 5). For the **superior** part of the femoral neck the highest tensile strain was found for walking at 6 km/h (second peak– 5885 με) followed by running at 9, 7and 8 km/h (5415, 4717 and 4673 με, respectively) (Fig 6). Notably, all resistance exercises induced lower tensile strain magnitudes compared to normal walking at 4 km/h. Overall, peak tensile principal strains at the superior part of the femoral neck ranged from 450 με (hip adduction at 40% RM) to 5885 με (6 km/h walking–second peak). In most exercises, tensile strains at the superior part were higher than in the inferior part except for hopping landing and propulsion (239% and 230%).

### Peak compressive strains

**Fig 8:**
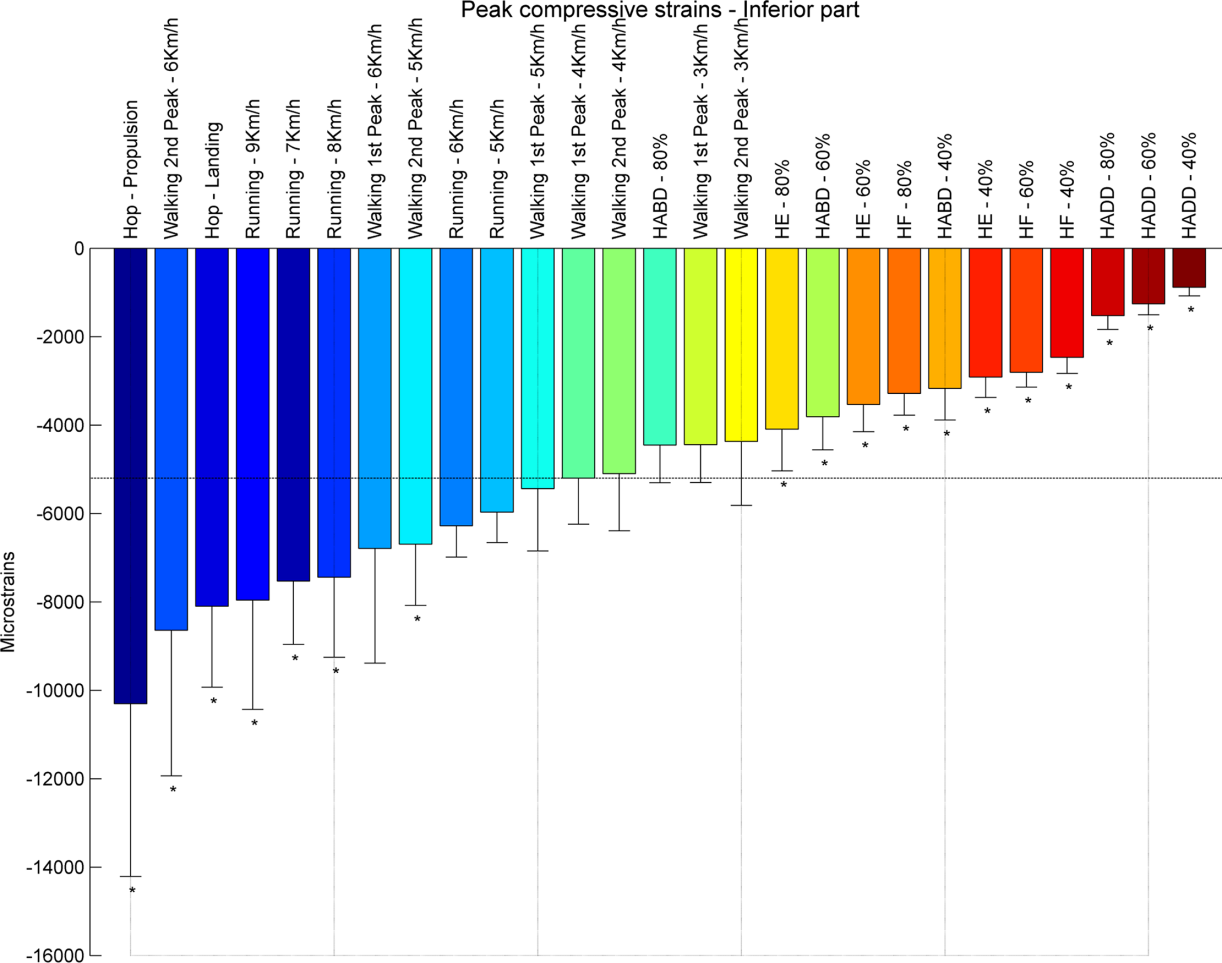
Ranking compression strains inferior part. Average peak compressive strains in μstrains (εμ) in the inferior part of the femoral neck ranked from left to right for the highest (blue) to the lowest strain (red). Asterisks denote the exercises with significantly different peak compressive strains compared to walking at 4 km/h (1^st^ peak) indicated by the horizontal line.

**Fig 9:**
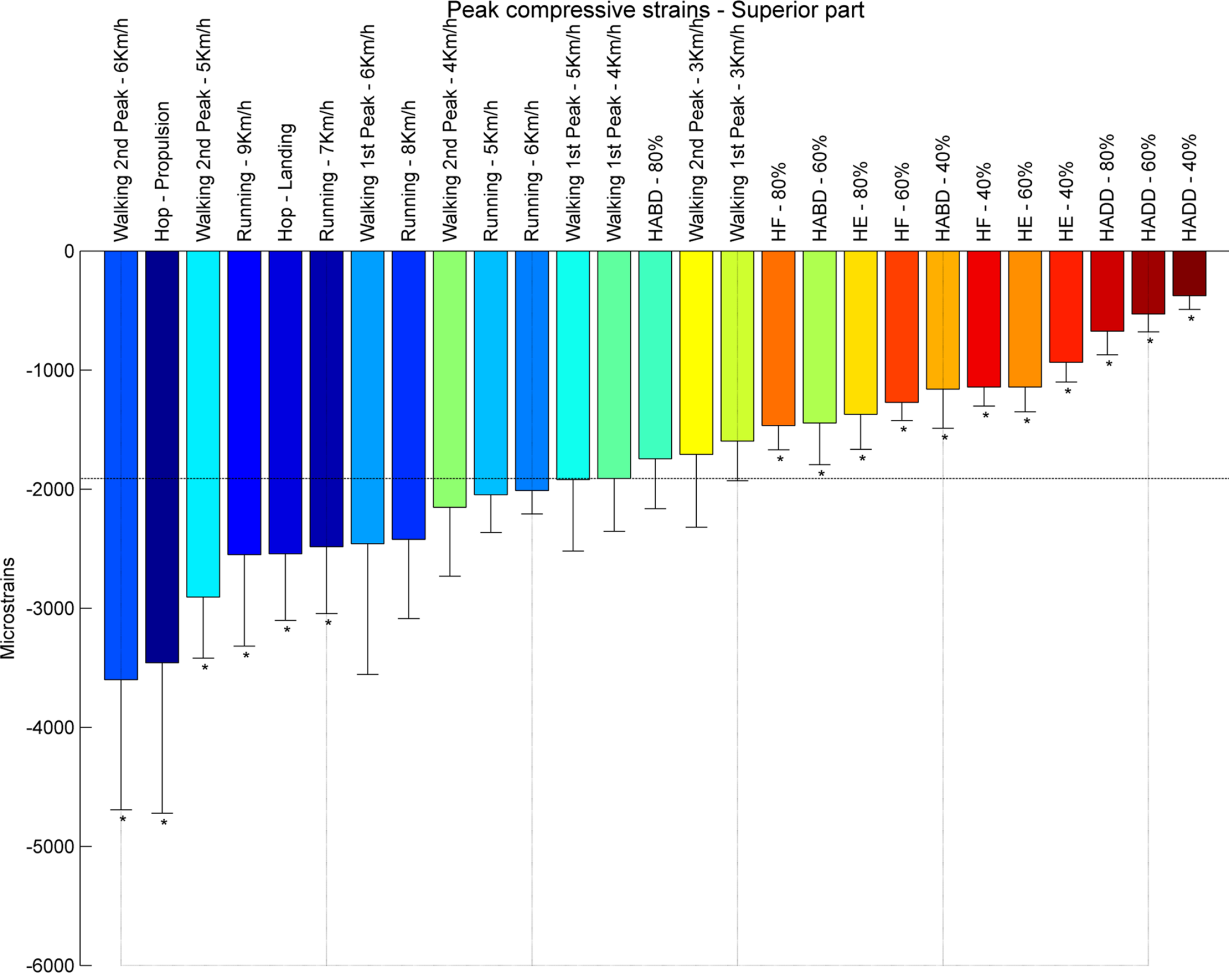
Ranking compression strains superior part. Average peak compressive strains in μstrains (εμ) in the s part of the femoral neck ranked from left to right for the highest (blue) to the lowest strain (red). Asterisks denote the exercises with significantly different peak compressive strains compared to walking at 4 km/h (1^st^ peak) indicated by the horizontal line.

At the **inferior** part of the femoral neck, propulsion phase of hopping (-10304 με) induced the highest compressive strains, followed by the second peak during walking at 6 km/h (-8644 με) (Fig 7). The compressive strain magnitudes for all resistance training exercises are lower compared to habitual walking at 4 km/h. Overall, peak compressive strains at the inferior part of the femoral neck range from -887 με (hip adduction at 40% RM) to -10304 (hopping–propulsion) (Fig 8). Hence, the **superior** part of the femoral neck is mostly compressed by fast walking (second peak) and hopping. The highest compressive strain was induced by the second peak during walking at 6 km/h (-3602 με), followed by hopping–propulsion (-3458 με), second peak of walking at 5 km/h (-2906 με) and running at 9 km/h (-2552 με) (Fig 9). Overall, peak compressive principal strains at the superior part of the femoral neck range from -377 με (hip adduction at 40% RM) to -3602 με. For all exercises, compressive strains at the inferior part were higher than in the superior part.

## Discussion

The current study aimed to assess the osteogenic potential at the femoral neck for different exercises by calculating and comparing peak compressive and tensile strains occurring at the inferior and superior part of the femoral neck in a group of postmenopausal women. Hopping, running and fast walking for most speeds results in significantly higher compressive and tensile strains at the femoral neck compared to walking at 4 km/h, possibly triggering an osteogenic effect at this particular area (Table 1).

**Table 1:** Exercises that have induced significantly higher (●) or lower (●●) (p < .05) hip contact or strains than 4 km/h walking (1^st^ peak) at the femoral neck (inferior and superior part).

	Hip Contact Force	Tensile Strains Inferior	Tensile strains Superior	Compression strains Inferior	Compression strains Superior
**Walking**					
3km/h - 1st peak	●●				
3km/h - 2nd peak	●●				
4km/h - 1st peak					
4km/h - 2nd peak					
5km/h - 1st peak					
5km/h - 2nd peak	●	●	●	●	●
6km/h - 1st peak	●	●			
6km/h - 2nd peak	●	●	●	●	●
**Running**					
5 km/h	●				
6 km/h	●				
7 km/h	●	●	●	●	●
8 km/h	●	●	●	●	
9 km/h	●	●	●	●	●
**Hopping**					
Landing	●	●	●	●	●
Propulsion	●	●	●	●	●
**Excercise**					
HF—40%	●●	●●	●●	●●	●●
HF—60%	●●	●●	●●	●●	●●
HF—80%	●●	●●	●●	●●	●●
HE—40%	●●	●●	●●	●●	●●
HE—60%	●●	●●		●●	●●
HE—80%	●●	●●		●●	●●
HABD—40%	●●	●●	●●	●●	●●
HABD—60%			●●	●●	●●
HABD—80%			●●		
HADD—40%	●●	●●	●●	●●	●●
HADD—60%	●●	●●	●●	●●	●●
HADD—80%	●●	●●	●●	●●	●●

Lack of in vivo strain data at the femoral neck during the exercises prohibits a direct comparison with the obtained strain results. However, the calculated femoral head displacements at peak hip loading during walking are small enough (< 3 mm) to be considered physiological [34,35] and can therefore serve as an indirect validation of the strains calculated in our study [30].

Not all exercises imposed significantly higher strains at the femoral neck than 4 km/h walking (Table 1), whereby mostly the resistance training exercises scored lower. Of all exercises tested in the study, hopping (landing and propulsion) resulted in the highest compressive and tensile strains at the inferior part of the femoral neck. Fast walking (5–6 km/h, 2^nd^ peak) resulted in higher compressive strains at the inferior and superior part and higher tensile strains at the superior femoral neck compared to walking at 4 km/h (1^st^ peak). However, only resistance training exercises at 80% RM induced similar strains at the femoral neck. At lower intensities than 80% RM the resistance training exercises induce significantly lower strains at the femoral neck compared to walking at 4 km/h (1^st^ peak). These results are partly in agreement with several clinical trials that suggest that a fast walking intervention program [13,36,37] and hopping (vertical/multidirectional) [38–40] can induce an increase in the femoral neck BMD in an elderly populations. Hence, in contradiction with previous studies [41,42] our results suggest no, or even a negative, effect of hip targeted resistance training exercises on the femoral neck BMD, except at intensities above 80% RM. Therefore these exercises alone seem to be unsuitable to increase or maintain the femoral neck BMD in people with a high risk of osteoporotic fracture.

Results shows that the largest strains occur in load bearing exercises such as walking, running and hopping. Interestingly, our results indicate that some exercises with similar HCFs, such as walking at 4 km/h (second peak) and HABD at 60% RM, can induce different strain distributions at the femoral neck. Whereas for hopping the HCF and strain ranking are high in both femoral neck areas, this is not the case for the second peak of walking which induce higher tensile strains in both the inferior and superior part and higher compression strains at the superior part of the femoral neck compared to their corresponding HCFs ranking (Figs 2–7). In contrast, exercises with muscle action alone seem not to result in similar strains. Most resistance training exercises impose significantly lower HCFs and strains than walking at 4 km/h (Table 1). It should be noted that the strain distribution due to the hip contact forces, tensile in the superior and compressive in the inferior part, is also modulated by the muscle recruitment strategy. Large muscles, such as the gluteal muscles, become active during the gait cycle to counteract the joint moment and affect the local strain distribution at the femoral neck by generating compressive strains in the superior and tensile strains in the inferior part. Overall, resulting strains are therefore reduced during walking and running. This might explains the difference between the first and the second peak of walking since gluteal muscles are merely active during the first peak. As a result the compression and tensile strains for the second peak are always higher compared to the first peak of walking. Therefore, our results suggest that high HCFs are strongly related to the strain distribution in the proximal part of the femur and are influenced by bending and torsion as a result of the local muscle action as well as HCFs. The latter may explain the lower tensile strains for the hip flexion/extension and hip abduction/adduction at 80% RM at the superior part of the femoral neck in our study compared to the values reported by Martelli et al.

The osteogenic potential of an exercise however depends not only on the strain ranking but also fracture risk needs to be taken into account when selecting training exercises. In linear FEA, the bone fraction that exceeds a specific threshold is often used as a definition of failure. The Pistoia criterion states that if 2% of the tissue exceeds an effective strain of 7000 μstrains (43) it is likely that a fracture will occur. Using this criterion, hopping would be considered as an exercise with a high risk of fracture in elderly and may therefore not be suitable as a training exercise.

### Limitations

A workflow combining musculoskeletal modeling and FEA was used in this study to adequately represent the mechanical environment including all muscle and reaction forces acting on the femur during various physical exercises. However, limitations are inherent to this approach. Calculation of muscle forces, and consequently joint forces, is subject to the model and optimization technique used. Static optimization has been found to satisfactorily reproduce muscle activation patterns during walking [43,44] and hopping [45], although the produced force magnitudes still remain invalidated due to the impracticability of in vivo data acquisition.

Other factors such as the model’s boundary conditions, material properties and geometry may affect our results in a certain amount. A CT-scan of the femur of a healthy 60 year old male was selected as a template to estimate the material properties of an elderly female population, given that the HU template was close to the average HU distribution of 9 post-menopausal elderly subjects normalized by their HU range. However, differences in material properties e.g. a lower BMD as present in an osteoporotic population, would exhibit higher strains due to the lower elastic modulus compared to a healthy population and may therefore alter our results. Alternatively, BMD of cadaveric samples could have been used [16,35,46]. However, given the frailty of these donors, material properties would not have been representative for the more active elderly population which are likely to have an influence on the BMD and material properties due to a more frequent mechanical loading.

In the current study, a femur with an identical geometry of the generic musculoskeletal model uniformly scaled to represent the subject’s anthropometry was used for all subjects in the FEA. As a result, geometrical variations in the proximal femur that will inherently affect the calculated strains for each subject, were not accounted for in the current study. However, this approach guarantees dynamic consistencies between the geometry of the musculoskeletal and FE model. Future research will therefore focus on the inclusion of the subject-specific geometry in both the musculoskeletal and FE models, as well as subject-specific material properties, loading and boundary conditions to yield more physiological strain estimates in this specific cohort.

Applying a failure criterion, hopping would be considered as an exercise with a high fracture risk. However, such criteria are depending on bone geometry, mesh, material properties and loading conditions and should be interpreted carefully given the inferior physical condition in the elderly [47]. Nevertheless, we feel that the conclusions based on relative comparisons of the strain magnitudes are still valid as long as volume, frequency and duration of each exercise and the training program is adequate. This also indicates the need for more clinical training studies on the effect of strains on the BMD at the proximal femur to determine the osteogenic potential of specific training exercises.

## Conclusion

The present study provides a comparative analysis of strain data occurring at the superior and inferior part of the femoral neck during potentially osteogenic exercises performed by healthy post-menopausal women. A comparison between the strain magnitudes and the peak HCFs for different exercises revealed that factors such as muscle recruitment strategy have an influence on the strain distribution especially at the superior part of the femoral neck. Since superior femoral neck frailness is related to elevated hip fracture risk [32,48–52], exercises such as fast walking and running can be highly recommended to stimulate this particular area to address femoral neck fragility. The use of hopping needs to be considered cautiously given the high strains and fracture risk reported. In conclusion, our results suggest that a training program including fast walking (above 5 km/h) and running exercises may increase or preserve the BMD at the femoral neck. Future work will address the inclusion of more subject-specific details in the musculoskeletal and FE models. Ultimately, a bone density adaptation model can be implemented to evaluate the osteogenic response over time and the efficacy of a specific training program in preventing bone loss at the femoral neck.

## Supporting information

S1 Table Results linear mixed models. (XLSX)
